# Effect of continuous nursing on quality of life of hemodialysis patients: A protocol for systematic review and meta-analysis

**DOI:** 10.1097/MD.0000000000024942

**Published:** 2021-03-26

**Authors:** Ling Yuan, Haitao Yuan, Qingchun Feng, Jinyu Zhao

**Affiliations:** a Tangshan Workers’ Hospital, Tangshan, Hebei Province; b The Medical Emergency Center of Tianjin Binhai International Airport; c Nankai Hospital of traditional Chinese Medicine Affiliated to Tianjin University of traditional Chinese Medicine, Tianjin, Tianjin Province; d Tangshan Union Medical College Hospital, Tangshan, Hebei Province, China.

**Keywords:** continuous nursing, hemodialysis, protocol, randomized controlled trial

## Abstract

**Background:**

Hemodialysis is a common treatment for patients with end-stage renal failure. Long-term hemodialysis will lead to a series of complications and reduce the quality of life of patients. At present, routine nursing is only limited to in-hospital patients, whereas continuous nursing is an extension of hospital nursing work, which can solve the follow-up health problems of some patients and meet the health needs of patients in their daily life. A number of clinical studies have shown that continuous nursing can improve the quality of life of hemodialysis patients, but there is a lack of evidence-based medicine. Therefore, the purpose of this study is to explore the effect of continuous nursing on the quality of life of hemodialysis patients through systematic evaluation.

**Method:**

The Cochrance Library, PubMed, Embase, Web of Science, CNKI, VP Information Chinese Journal Service Platform (VIP), and Wanfang will be searched by computer. In addition, Baidu academic and Google academic are searched manually, and all randomized controlled trials on the effects of continuous nursing on the quality of life of hemodialysis patients are collected. The retrieval time is of the establishment of the database to January 31, 2021. Two evaluators screens, extract and evaluate the literature, and the data are analyzed by RevMan5.3 software.

**Result:**

The purpose of this study is to evaluate the effect of continuous nursing on the quality of life of hemodialysis patients by the MOS item short from health survey, exercise of self-care agency, and the incidence of complications.

**Conclusion:**

This study will provide reliable evidence for the application of continuous nursing in hemodialysis patients.

**OSF Registration number:**

DOI 10.17605/OSF.IO/HZKUA

## 1 Introduction

Chronic kidney disease has become one of the public health problems that endanger global health.^[1]^ With the progress of the disease, it will eventually become end-stage renal disease (ESRD).^[2,3]^ At present, the main treatment of ESRD patients is hemodialysis, which takes a long time and affects the quality of life of patients from physical, psychological and social aspects.^[4]^ When ESRD patients are undergoing hemodialysis in the hospital, nurses give routine care to inform the relevant matters needing attention, but patients spend most of their time outside the hospital, so it is difficult to solve the sudden situation of the disease. At the same time, due to the lack of supervision, poor compliance with drugs or diet may occur, which not only brings difficulties to treatment, but also brings greater economic burden to patients.^[5]^

Continuous care refers to a series of nursing measures for patients in different health care places (such as hospitals, communities, families) or the same health care places (such as different departments in a hospital). So that patients receive different levels of continuous care.^[6]^ In 2011, the extended nursing service project was formally incorporated into the research field of the Ministry of Health,^[7]^ and then put forward that continuing nursing is the key task of the “Twelfth five-year Plan” period,^[8]^ which shows the importance of continuing nursing. Luo et al^[9]^ found that maintenance hemodialysis patients have a strong demand for continuous care, so it is suggested to provide targeted extended nursing services to improve the quality of life of hemodialysis patients. At present, many studies have shown that the application effect of continuous nursing in hemodialysis patients is significant, and the incidence of complications is low, but there are differences in continuous nursing programs and curative effect evaluation in various clinical studies, and the results are uneven. To a certain extent, it affects the reliability of the research results and the promotion of continuous nursing, so this study adopts the method of systematic evaluation. To further clarify the effect of continuous nursing on the quality of life of hemodialysis patients, to provide a reliable reference for hemodialysis patients to receive standardized continuous nursing.

## 2 Methods

### 2.1 Protocol register

This protocol of systematic review and meta-analysis has been drafted under the guidance of the preferred reporting items for systematic reviews and meta-analyses (PRISMA-P). Moreover, it has been registered on opening science framework (OSF) on November 25th, 2020 (registration number: DOI 10.17605/OSF.IO/HZKUA).

### 2.2 Ethics

Since this is a protocol with no patient recruitment and personal information collection, the approval of the ethics committee is not required.

### 2.3 Eligibility criteria

#### 2.3.1 Types of studies

We will collect the published randomized controlled trials on continuous nursing intervention for hemodialysis patients at home and abroad, regardless of blinding, publication status, region, but Language will be restricted to Chinese and English.

#### 2.3.2 Research object

The nationality, race, age, sex, and course of disease of the patients undergoing hemodialysis are not limited.

#### 2.3.3 Intervention measures

The control group adopt routine nursing, whereas the experimental group adopt continuous nursing on the basis of the control group, which includes setting up a special extended nursing group and making a detailed out-of-hospital nursing plan, including helping patients and their families understand the disease correctly, cultivate good eating habits, guide reasonable exercise, and care of internal fistula, and so on, and conduct regular telephone or WeChat follow-up to know the patient's condition.

#### 2.3.4 Outcome index

1. Primary outcome: the MOS item short from health survey
2. Secondary outcomes: exercise of self-care agency; complication rate.

### 2.4 Exclusion criteria

1. Repeatedly published articles.
2. The literature is nonrandomized controlled trial, such as review, case report, lecture, and so on.
3. Articles in which the published literature is an abstract or with incomplete data. And the data cannot be obtained after contacting the author.
4. There are obvious errors in the data.
5. Literature whose outcome index is not clear.
6. Literature that randomizes or allocates concealment as a high risk of bias.^[10]^

### 2.5 Retrieval strategy

CNKI, Wanfang Data, VIP, PubMed, Cochrane Library, Embase, Web of Science and other databases will be searched by computer. The key Chinese words are “hemodialysis,” “renal dialysis,” and “continuous care”, and the English key words are “Hemodialysis,” “Renal Dialyses,” “Continuum of Care,” and “Continuous care.” Both Chinese and English are searched by the way of subject words combined with free words, and the search time is from the establishment of the database to January 31, 2021. In addition, a manual search is conducted in Baidu academic and Google academic to collect all the randomized controlled trials of the effects of continuous care on the quality of life of hemodialysis patients. The PubMed is shown in Table 1.

**Table 1:** Retrieval strategy of PubMed.

Number	Search terms
#1	Renal Dialysis[Mesh]
#2	Dialyses, Renal[Title/Abstract] OR Renal Dialyses[Title/Abstract] OR Hemodialysis[Title/Abstract] OR Hemodialyses[Title/Abstract] OR
#3	Hemodialysis, Home[Mesh]
#4	Renal Dialysis, Home[Title/Abstract] OR Dialyses, Home Renal [Title/ Abstract] OR Dialysis, Home Renal[Title/Abstract] OR Home Renal Dialysis[Title/Abstract]
#5	Continuity of Patient Care[Mesh]
#6	Care Continuity, Patient[Title/Abstract] OR Patient Care Continuity [Title/Abstract] OR Continuum of Care[Title/Abstract] OR Care Continuum[Title/Abstract] OR Continuity of Care[Title/Abstract] OR Care Continuity[Title/Abstract] OR continuing nursing[Title/Abstract] OR Transitional Care[Title/Abstract] OR Continuous care[Title/Abstract]
#7	#1 OR #2 OR #3 O #4
#8	#5 OR #6
#9	#7 AND #8

### 2.6 Data screening and extraction

**Figure 1:**
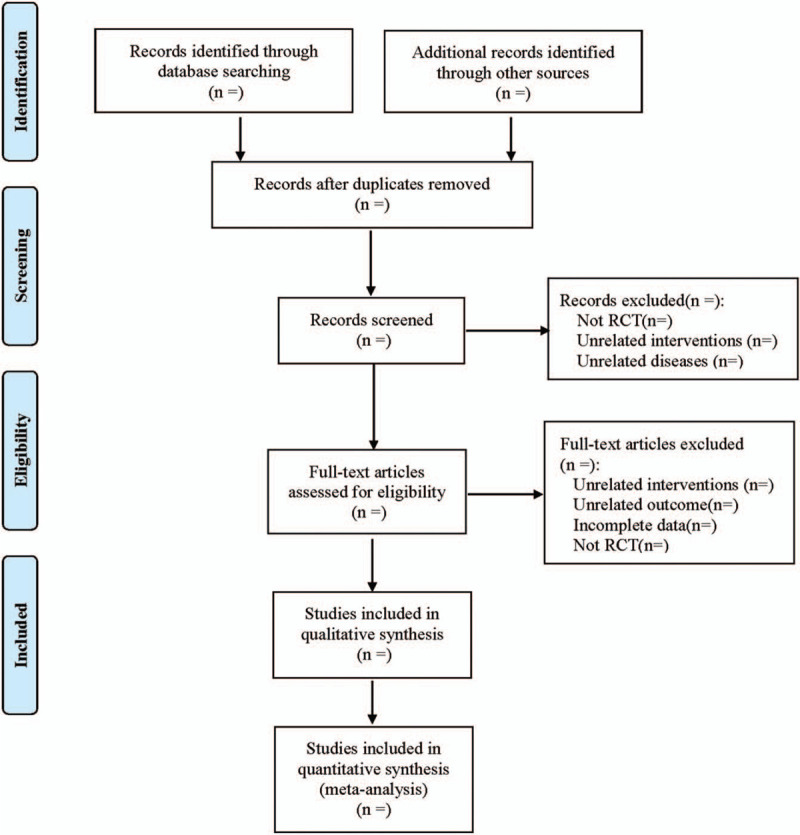
Flow diagram.

Refer to the method recommended by the Cochrane collaboration Network system evaluator Handbook version 5.0, and follow the PRISMA flow chart. According to the inclusion and exclusion criteria, the 2 researchers independently screen the literature through the document management software, cross-check the results, and ask the third researcher whether there are differences. Excel 2013 will be used to extract relevant information, including: basic information (such as title, first author, year of publication, country, sample size, age, among others); intervention measures; outcome indicators; risk bias assessment factors in randomized controlled trials. The literature screening process is shown in Figure 1.

### 2.7 Literature quality evaluation

The risk of bias for each eligible study will be assessed by 2 researchers respectively according to the Cochrane Collaboration's tool including 7 terms. According to these criteria (random sequence generation, allocation concealment, blinding, incomplete data, selective result reports, and other bias), risk of bias is classified into the following levels: unclear, low, and high risk of bias. Any divergences will be solved through discussion by a third researcher.

### 2.8 Statistical analysis

#### 2.8.1 Data analysis and processing

The RevMan 5.3 software is used for statistical analysis. For dichotomous variables, relative risk (RR) is used for statistics. For continuous outcomes, weighted mean difference is selected when the tools and units of measurement indicators are the same, standardized mean difference is selected when the tools and units of measurement indicators are different, and all the above were represented by effect value and 95% confidence interval (CI). Heterogeneity is determined by *χ*^*2*^ and *I*^*2*^ values. If (*P* ≥ .1, *I*^*2*^ ≤ 50%) indicated low heterogeneity. Fixed effect model is used for meta-analysis. If (*P < *.1, *I*^*2*^ > 50%) indicated heterogeneity among studies, and the source of heterogeneity will be explored through subgroup analysis. If there is no obvious clinical or methodological heterogeneity, it will be considered as statistical heterogeneity, and the random-effect model will be used for analysis. Descriptive analysis is used instead of meta-analysis if there is significant clinical heterogeneity between the 2 groups and subgroup analysis is not available.

#### 2.8.2 Dealing with missing data

If there are missing data in the article, contact the author through email to the relevant information. If the author cannot be contacted, or if the author has lost the relevant data, a descriptive analysis is performed, not a meta-analysis.

#### 2.8.3 Subgroup analysis

According to the different course of continuous nursing, the subgroup analysis will be carried out, and the subgroup analysis will be carried out according to the different ways of continuous nursing.

#### 2.8.4 Sensitivity analysis

To determine the stability of outcome indicators, sensitivity analysis was used to analyze each outcome indicator.

#### 2.8.5 Assessment of reporting biases

Funnel plots are used to assess publication bias if no <10 studies are included in an outcome measure. Moreover, Egger and Begg test are used for the evaluation of potential publication bias.

#### 2.8.6 Evidence quality evaluation

The Grading of Recommendations Assessment, Development, and Evaluation (GRADE) will be used to assess the quality of evidence. It contains 5 domains (bias risk, consistency, directness, precision, and publication bias). And the quality of the evidence will be rated as high, moderate, low, and very low.

## 3 Discussion

Continuous nursing is a new nursing model, which pays attention to the continuity and integrity of patients’ health guidance, mainly the continuation of patients’ life from hospital to family, and the guide is not interrupted because of the transfer of places. Continuous nursing includes 2 key factors: individual health service and nursing service time continuation, which can be divided into 3 types: the continuation of information, the continuation of management, and the continuation of relationship.^[11]^ There are many modes of continuous care, and studies have confirmed that guided nursing model,^[12]^ APN continuing nursing model,^[13]^ evaluation and care of the elderly resource model,^[14]^ and continuous nursing model^[15]^ have achieved positive results in clinical application. Rezamand et al^[16]^ found that continuous nursing can improve the self-care ability of patients with heart failure. Siegmann et al^[17]^ showed that the overall health status of patients with diabetes after continuous care was better than that of the routine nursing group. Other studies have shown that continuous care can reduce the rate of rehospitalization and the risk of rehospitalization.^[13,18]^ Continuous nursing is the connection of hospital nursing plan, which aims to improve patients’ health and quality of life, and can also promote the establishment of a good relationship between doctors and patients.

ESRD patients have dialysis treatment every week, which cannot be completed at one time by hospitalization. Zhang et al^[19]^ found that the level of self-management ability and diet and exercise compliance of maintenance hemodialysis patients is relatively low, and simple health guidance cannot improve the living standards of patients. Therefore, medical staff can carry out effective continuous nursing programs for hemodialysis patients, such as vascular access nursing education and guidance, drug use education and guidance, diet and nutrition nursing education, psychological counseling and emotional support, exercise guidance, to improve patients’ self-management level and quality of life.^[20]^ Borji et al^[21]^ found that continuous nursing can improve the quality of life of patients, but has no significant effect on their blood pressure. Otaghi et al^[22]^ through clinical trials, have found that continuous nursing intervention can improve the sleep quality of hemodialysis patients. Rahimi et al^[23]^ found that continuous nursing has a positive effect on anxiety, depression, and stress of patients with hemodialysis, which is highly practical.

At present, there are many studies on continuous nursing intervention in hemodialysis, but some studies have some deficiencies in design, which cannot provide a good evidence-based basis for the implementation of extended nursing in hemodialysis patients. Therefore, this study analyzes the existing RCTs test of continuous nursing intervention in hemodialysis patients, objectively evaluates the effect of continuous nursing on the quality of life of hemodialysis patients, and provides evidence-based medical evidence for further development of continuous nursing in hemodialysis patients. This study also has some limitations, because there is no standard scheme for continuing nursing, there may be some clinical heterogeneity. And this study only searches English and Chinese literature, and may ignore studies or reports in other languages, and there is a certain publication bias.

## Author contributions

**Data collection:** Ling Yuan and Haitao Yuan

**Data curation:** Ling Yuan, Haitao Yuan.

**Funding acquisition:** Qingchun Feng, Jinyu Zhao.

**Funding support:** Qingchun Feng and Jinyu Zhao

**Resources:** Jinyu Zhao and Haitao Yuan

**Software operating:** Qingchun Feng and Jinyu Zhao

**Software:** Qingchun Feng, Jinyu Zhao.

**Supervision:** Haitao Yuan.

**Writing – original draft:** Ling Yuan and Haitao Yuan

**Writing – review & editing:** Ling Yuan, Qingchun Feng and Jinyu Zhao
